# A North-European perspective challenges the UK NICE system for resource allocation

**DOI:** 10.3332/ecancer.2010.ed7

**Published:** 2010-05-17

**Authors:** Silvia Camporesi

**Affiliations:** European School of Molecular Medicine & University of Milan

Economist and ethicist in waiting Erik Nord (Norwegian Institute Public Health Professor Health Economics, Oslo) -as he defined himself- gave a North-European perspective on the UK model of allocating resourceswith regard to expensive cancer drugs, at the fifth international bioethics conference ‘Priorities in Health: can we make better decisions?’ conference at Harvard Universisty (April 22-23, 2010).

The UK health care system is regulated by the ‘National Institutes for Health and Clinical Excellence’ (‘NICE’), which was set up in 1999 to ensure that everyone, regardless of where they live in England and Wales, has equal access to medical treatments and high quality care from the National Health Service (‘NHS’). NICE makes recommendations to the NHS on new and existing medicines, treatments and procedures, and on treating and caring for people with specific diseases and conditions (http://www.nice.org.uk/)

Since its birth, NICE raison d’etre has been cost-effectiveness analysis (CEA) of health technology interventions. Typically in health-care economics, the CEA is expressed in terms of a ratio where the denominator is a gain in health from a measure (years of life, premature births averted, sight-years gained) and the numerator is the cost associated with the health gain. The most commonly used outcome measure is Quality-Adjusted Life Years (QALY), which is based on the number of years of life that would be added by the intervention. Each year in perfect health is assigned the value of 1.0 down to a value of 0.0 for death. If the extra years would not be lived in full health, then the extra life-years are given a value between 0 and 1 to account for this (Nord et al, Health Economics 2010).

NICE requires CEA of selected medical technologies, as part of the basis for their coverage recommendations to the NHS, and it applies a criterion of around £30,000 per QALY or less. In other words, a medical treatment is not approved by NICE if it exceeds the maximum cost of £ 30,000 or less per QALY gained. Yet even in the UK there are increasing complaints about denial of expensive but marketed treatments for life-threatening conditions, such as cancer and dementia.

As a result, NICE has implemented a compassionate care exception to the £30,000 per QALY criterion on the basis of the following reasons:

- when QALYs/EQ-5D do not capture quality of life (‘QoL’) gains fully, where EQ-5D is a standardised instrument for use as a measure of health outcome that provides a simple descriptive profile and a single index value for health status (http://www.euroqol.org)
- when the treatment is considered to have a long term value in ‘innovation’;
- where there is no existing alternative treatment.

Not all kinds of reasons, tough, are accepted by NICE to make exceptions. Traditionally rejected reasons have been the following two: the former being based on the rule of rescue (according to which priority to treatment goes to the ones who are worse off) and on rare conditions. There are also motivations which have not been considered at all by NICE, as providing equal rights to individuals (who start from different health conditions) to realise their health potentials.

Both types of reasons (traditionally rejected or not considered) have raised issues of fairness in the use of QALYs as the only measure for a cost effective analysis in health care. A recent paper coauthored by Erik Nord, Norman Daniels (Department of Population and International Health, Harvard School of Public Health) and Mark Kamlet (Heinz School of Public Policy and Management, Carnegie Mellon University, Pittsburgh) addressed the main ethical issues that in the authors’ opinion arise from the use of QALYs in health economics (Nord et al, Value Health 2009).

To begin with, as standard QALYs express value of a treatment in terms of ex ante self-interest, they are meant to express the personal utility of health outcomes as judged before the health outcome actually instantiates, and on the basis of the “average value” which would be expressed by the general public from behind a ‘veil of ignorance’ about future health (so-called “decision utility”). There are, however, possible alternatives, as highlighted by Nord in his talk. First, the so-called “experiences utility”: health state utilities may in principle be elicited ex post rather than ex ante, i.e. from people who have had direct experience with the health states that are the object of the valuation. Second, QALYs may be constructed to express society’s valuation of health outcomes when not only self-interest, but also concerns for fairness are taken into account. Third, as the work of Afam Oliver has shown, one of the major foundational issues around QALYs is that their use is based on the Standards Gamble method, which in turn rests on the -at least debatable!- assumption that people are rational actors (Oliver A, J Economic Psych 2003; Oliver A, J Publ Health Meth 2003).

Other authors have also recently criticises the QALYs approach on the basis of fairness concerns (Drummond et al, Value Health 2009; Sulmasy et al, 2007). While the information provided by the QALYs is certainly a useful information, according to these authors setting priorities in health care must be based on a wider set of considerations. A cost-per-QALY ratio indicates the cost-effectiveness of an intervention. As such, the ratio is a measure of efficiency, rather than of “fairness.” Standard QALYs fail to take into account distributive concerns, e.g. the relative priority given to individuals of different levels of current health, or different capacity to benefit in terms of life expectancy or health-related quality of life. In decisions about resource allocation across patient groups, concerns for fairness may cause social resource allocation preferences to deviate considerably from the ranking that consideration of costs per QALY alone would suggest.

How could the QALY model be modified in practice in order to incorporate a wider set of considerations? Drummond and coauthors sketch out some possible strategies which emerged from the ISPOR Development Workshop on “Moving the QALY Forward: Building a Pragmatic Road” (Drummond et al, Value Health 2009). One modification could be to count as equal to 1 all gained life-years, even if they are in less than full health, as long as they are good enough to be desired by the individuals concerned. A second modification could be to place less weight on the duration of health benefits in comparisons of programs for patients with different life expectancies. Finally, a third modification would be to add explicit equity weights to the quality of life weights of the conventional QALY model.

All these possible modifications do not touch, though, upon a more profound problem of the use of QALYs in health economics (Nord et al, Value Health 2009). According to Nord, Daniels and Kamlet, reflections on the lack of fairness in the use of QALYs should lead us to raise the question as to whether, in societal valuations of health programs, there may be a need to distinguish between valuations of preventive and curative programs. As conventional QALYs reflect the general public’s ex ante judgments of the undesirability of different health states, one might say that they primarily speak to the challenges of setting priorities between preventive programs, and that when it comes to valuing and comparing interventions and treatment programs for people with different degrees of severity of illness and different potentials for health, more sophisticated models may berequired.

In which contexts could adjusted QALYs be used? Potentially they could inform various types of decisions. Traditionally, they have been used to inform broad resource allocation decisions among groups in the population, but they could also be used to inform the choice of treatment for individual patients or patient groups, as within the context of a private health plan (Drummond et al, Value Health 2009). It is important to recognize these various potential uses of QALYs because this is one source of the disagreement over whose values (members of the general population? Patients? Other subgroups?) should be used in constructing QALYs.

Cancer care costs money, and deciding how to allocate resources to treat cancer is inevitably a moral as well as a political act. If the rationing of oncologic care is necessary, as it seems, then the decision making process about the allocation of resources must be engaged through a deliberative democracy process. As Daniel Sulmasy -recently appointed member by President Brack Obama of the new Presidential Commission for the Study of Bioethical Issues, PCSBCI- society can only maintain respect for the dignity of each individual patient, while acknowledging that resources are limited, if health care rationing decisions are made through open, public, participatory processes that involve oncologists, patients, pharma and all the different stakeholders (Sulmasy, J Clin Onc 2007).

Are ‘rationing’ and ‘CEA’ necessarily bad words? According to some, they are. Title VIII of the American Recovery and Reinvestment Act of 2009 authorizes the expenditure of $1.1 billion to conduct research comparing “clinical outcomes, effectiveness, and appropriateness of items, services, and procedures that are used to prevent, diagnose, or treat diseases, disorders, and other health conditions”. Although cost is not mentioned explicitly in the comparative effectiveness legislation, the American College of Physicians and others have called for cost-effectiveness analysis to be on the agenda for comparative effectiveness research (CER). This approach has been harshly criticised from those who view it as the first step in health care rationing by the government, and that consider ‘rationing’ necessarily a bad thing (Weinstein et al, NEJM 2010).

Others have a different opinion. As argued by Robert Truog (Professor of Medical Ethics, Anaesthesiology & Pediatrics at Harvard Medical School and a Senior Associate in Critical Care Medicine at Children’s Hospital Boston) , the “R” word is not by itself a bad word (nor is cost-effective analysis), and we have to start thinking that no health care system in the world is free from the necessity of rationing. With Truog’s words, “The choice is not between health care rationing and some undefined alternative, since there is no alternative” (Truog, NEJM 2009).

So, what are we left to do? Actually, we have a lot of work to do. We have to decide which principles we will use to ration health care. We have to choose if we are content with a system based only on QALYs, as the UK NICE system, or with a system as the US, which has “traditionally rationed health care in the same way we ration expensive cars: those who can afford to pay for them are those who can have them” (Truog, NEJM 2009). Also, we have to challenge the assumptions that ‘new’ (drug) means ‘better’, or that a marketing authorisations means that all the drugs available in the market are equally effective. Plus, we need to challenge the private model of cost and price, both of which are highly elastic. At least, we should start thinking that it is possible to challenge things, instead of taking the ‘status quo’ for granted and be happy with it.

And, if we are not, we will have to choose how best to incorporate in our decisions about allocation of scarce resources other principles, such as those rooted in considerations of fairness and equity. Not an easy task. But someone has to do it!
